# Supervised fine-tuning of pre-trained antibody language models improves antigen specificity prediction

**DOI:** 10.1371/journal.pcbi.1012153

**Published:** 2025-03-31

**Authors:** Meng Wang, Jonathan Patsenker, Henry Li, Yuval Kluger, Steven H. Kleinstein

**Affiliations:** 1 Program in Computational Biology and Bioinformatics, Yale University, New Haven, Connecticut, United States of America; 2 Program in Applied Mathematics, Yale University, New Haven, Connecticut, United States of America; 3 Department of Pathology, Yale School of Medicine, New Haven, Connecticut, United States of America; 4 Department of Immunobiology, Yale School of Medicine, New Haven, Connecticut, United States of America; Utrecht University, NETHERLANDSKINGDOM OF THE

## Abstract

Antibodies play a crucial role in the adaptive immune response, with their specificity to antigens being a fundamental determinant of immune function. Accurate prediction of antibody-antigen specificity is vital for understanding immune responses, guiding vaccine design, and developing antibody-based therapeutics. In this study, we present a method of supervised fine-tuning for antibody language models, which improves on pre-trained antibody language model embeddings in binding specificity prediction to SARS-CoV-2 spike protein and influenza hemagglutinin. We perform supervised fine-tuning on four pre-trained antibody language models to predict specificity to these antigens and demonstrate that fine-tuned language model classifiers exhibit enhanced predictive accuracy compared to classifiers trained on pre-trained model embeddings. Additionally, we investigate the change of model attention activations after supervised fine-tuning to gain insights into the molecular basis of antigen recognition by antibodies. Furthermore, we apply the supervised fine-tuned models to BCR repertoire data related to influenza and SARS-CoV-2 vaccination, demonstrating their ability to capture changes in repertoire following vaccination. Overall, our study highlights the effect of supervised fine-tuning on pre-trained antibody language models as valuable tools to improve antigen specificity prediction.

## Author summary

Antibodies are vigilant sentinels of our adaptive immune system that recognize and bind to targets on foreign pathogens, known as antigens. This interaction between antibody and antigen is highly specific, akin to a fitting lock and key mechanism, to ensure each antibody precisely targets its intended antigen. Recent advancements in language modeling have led to the development of antibody language model to decode specificity information in the sequences of antibodies. We introduce an additional step of supervised fine-tuning to enhance the accuracy of antibody language models in predicting antibody-antigen interactions. By training these models on large datasets of antibody sequences, we can better predict which antibodies will bind to important antigens such as those found on the surface of viruses like SARS-CoV-2 and influenza. Moreover, our study demonstrates the potential of the models to analyze complex immune system data, offering new insights into how our bodies respond to vaccination. These findings have significant implications for vaccine design, as accurate prediction of antibody specificity can guide the development of more effective vaccines.

## Introduction

Recent advancements in machine learning (ML) have revolutionized the field of antibody research, holding promise to enable high-throughput prediction of antigen specificity, a critical challenge in immunology and therapeutic antibody design. Historically, classical approaches such as docking simulations [1,2], which model physical interactions between antibodies and antigens, have been widely used for predicting binding specificity. While providing valuable structural insights, these methods are often too computationally intensive for high-throughput applications. In contrast, ML-based methods have emerged as scalable and efficient alternatives, capable of analyzing large datasets quickly while maintaining predictive accuracy and generalizability [3]. Among ML methods, deep learning-based approaches have shown promise in applications such as structure prediction and binding affinity estimation [4–8]. Antibody language models are specialized deep learning architectures trained on vast datasets of antibody sequences [9–16]. By leveraging techniques such as masked language modeling and attention mechanisms [17,18], these models effectively capture the complex sequence-structure-function relationships inherent in antibodies [19,20], achieving high predictive accuracy in tasks such as binding affinity, viral neutralization, and thermostability prediction [21–23]. Moreover, they offer a powerful framework for analyzing and interpreting large-scale antibody repertoire sequencing data [15], shedding light on the molecular mechanisms underlying immune system function and dysfunction.

Transfer learning, a cornerstone of modern machine learning, has emerged as a powerful paradigm for leveraging knowledge from one domain to improve performance in another. In the context of language models, transfer learning involves pre-training a neural network on a large dataset in a source domain and then fine-tuning it on a smaller dataset in a target domain, where labeled data may be scarce [24,25]. This approach capitalizes on the transferability of learned representations across related tasks or domains, enabling models to capture generic features that are transferable while adapting to task-specific nuances during fine-tuning without requiring extensive computational resources or labeled data. In the realm of antibody language models, fine-tuning offers a promising avenue for enhancing predictive accuracy and generalization across diverse antigen-specificity prediction tasks [15,16]. Individual antibody language models differ in their unique architectures, training datasets, and pre-training objectives, which may influence their ability to generalize at specific tasks.

In this study, we investigated the efficacy of supervised fine-tuning of pre-trained antibody language models in predicting binding specificity to two key antigens: the SARS-CoV-2 spike protein and influenza hemagglutinin. By fine-tuning pre-trained models on labeled data specific to these antigens, we aimed to enhance predictive accuracy and generalization across diverse antibody sequences. We further applied the fine-tuned models to BCR repertoire data for influenza and SARS-CoV-2 vaccination to investigate their ability to capture changes for vaccination response.

## Results

### Fine-tuning antibody language models for specificity prediction

**Fig 1:**
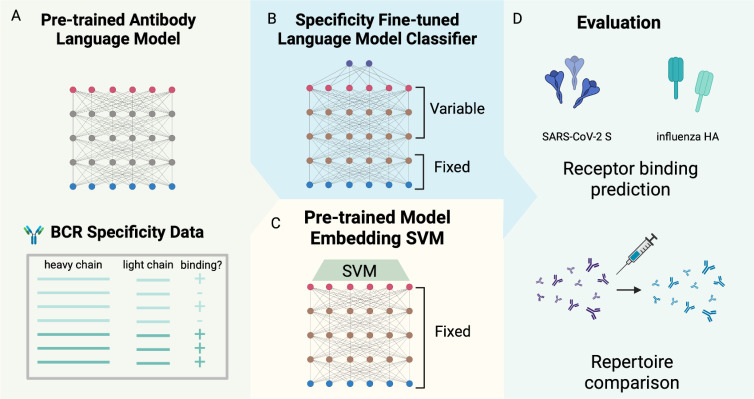
Fine-tuning antibody language model on receptor specificity prediction tasks. (A) Pre-trained antibody language model embeddings are used to train classifiers for SARS-CoV-2 spike protein and influenza hemagglutinin specificity prediction. (B) The pre-trained antibody language models were fine-tuned on the last three layers for specificity classification tasks, and (C) Supervised support vector machine classifiers were specifically used to train pre-trained model embeddings of BCR sequences. (D) The performance of the classifiers was evaluated on the cross-validation accuracy of receptor specificity prediction and comparison between longitudinal time points of repertoire datasets for SARS-CoV-2 and influenza vaccination. Created with Biorender.

To investigate the effect of supervised fine-tuning on predicting BCR specificity, we fine-tuned the last three layers of four pre-trained antibody language models, including antiBERTy [9], antiBERTa2 [11], BALM-paired [16], and ft-ESM2 [16] (Table 1), for binary binding status classification for SARS-CoV-2 spike (S) protein and influenza hemagglutinin (HA) (Fig 1). For performance comparison, we also trained supervised SVM on pre-trained model embeddings on the same task and data.

**Table 1:** Parameters of publicly available pre-trained antibody language models.

Model	Parameters	Hidden Size	Intermediate Size	Attention Heads	Layers
antiBERTy	25.76M	512	2048	8	8
antiBERTa2	202.64M	1024	4096	16	16
BALM-paired	303.92M	1024	4096	16	24
ft-ESM2	652.36M	1280	5120	20	33

In terms of specificity data, we collected in total 15,539 and 5,514 paired full-length BCR sequences for S protein and HA fine-tuning classification tasks, respectively, from publicly available datasets with binding and donor/study labels (Table 2). To balance the class, we sampled S protein non-binding sequences from pre-pandemic B cell repertoires described in [31], and HA non-binding sequences from influenza vaccine non-responsive B cells [30]. The sampled non-binding sequences have similar V, J gene usage, CDR3 length, and somatic hypermutation frequency distributions with the binding class (S1 and S2 Figs).

**Table 2:** Data sources for antigen-specific antibody sequence and vaccination related BCR repertoire.

Antigen	Data source	Type	Description	# Receptors
Influenza HA	IEDB [26], downloaded on Dec 2023	Sequence	Public database with curated BCR and epitope information, extracted human BCR sequences to influenza HA	113 (all binding)
Influenza HA	Turner 2020 [27]; McIntire 2024 [28]	Sequence	Human monoclonal antibodies sequences tested for binding to 2018 QIV HA	1,551 (all binding)
Influenza HA	Wang Y. 2023 [29]	Sequence	Curated database of human monoclonal antibodies to influenza HA	1,311 (861 binding)
Influenza HA	Wang M. 2023 [30]	Sequence	Influenza vaccination non-responsive BCR sequences	2,539 (all controls)
Influenza HA	Wang M. 2023 [30]	Repertoire	Single-cell BCR sequencing on patients receiving seasonal influenza vaccination	87,230
SARS-CoV-2 S	Wang M. 2024 [31]	Sequence	Collected public databases of human monoclonal antibodies to SARS-CoV-2 spike protein	15,539 (8658 binding)
SARS-CoV-2 S	Kim 2022 [32]	Repertoire	Single-cell BCR sequencing on patients receiving SARS-CoV-2 mRNA vaccine	164,252

To evaluate the fine-tuned classifiers and the pre-trained model embedding classifiers, we used four-fold cross-validation (CV) with non-overlapping donors/studies between each train-test split and evaluated the performance of the models on the test split. Within the training split of each fold, we also performed hyperparameter selection for fine-tuning by further splitting a validation set (33%) from the train set or performing another three-fold cross validation to train the pre-trained embedding SVM (S3 and S4 Figs).

### Supervised fine-tuning improves specificity prediction performance

**Fig 2:**
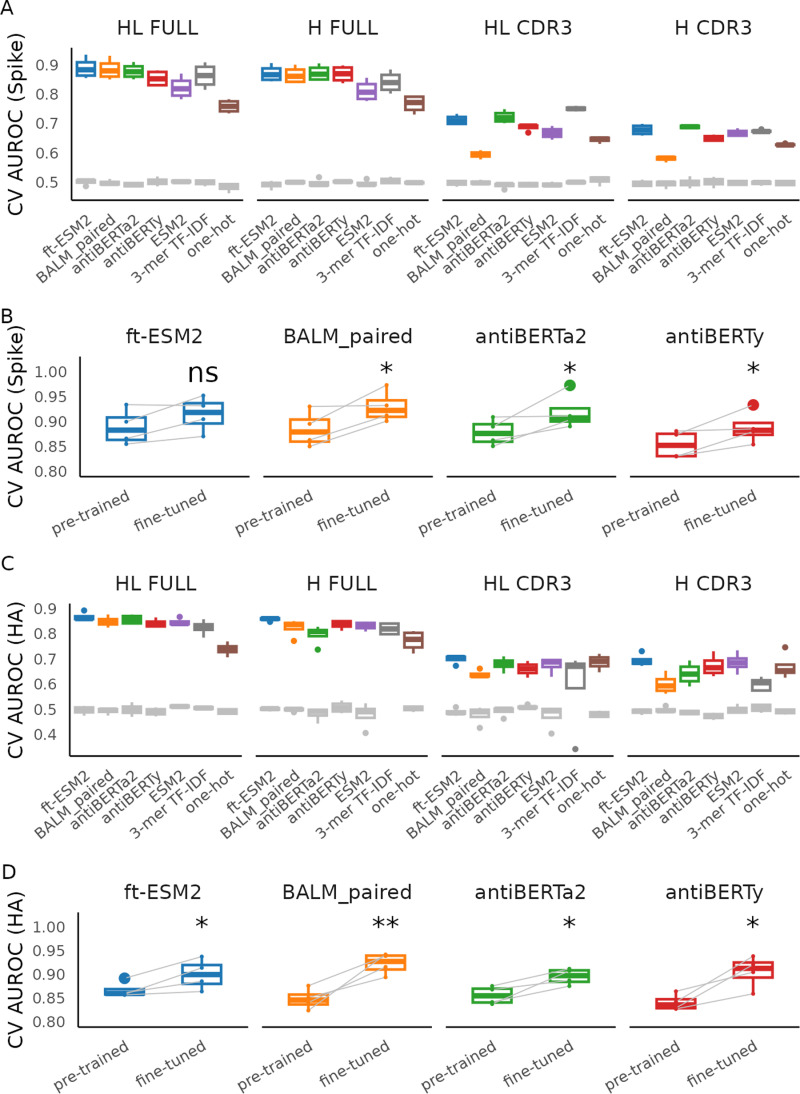
Specificity prediction performance for pre-trained model embedding SVM and fine-tuned antibody language models. The box plots display the 4-fold cross-validation AUROC for predicting binding to different antigens: (A) SARS-CoV-2 S protein and **(C)** influenza HA. The gray box plots denote the random baseline achieved by training on shuffled labels. Panel **(B)** compares the CV AUROC of the pre-trained embedding-based SVM model with fine-tuned language models for SARS-CoV-2 S protein, while panel **(D)** presents this comparison for influenza HA. Each line indicates test performance for an individual CV fold. Note that ft-ESM2 is a fine-tuned version of ESM2 with weights optimized on BCR sequence masked language model task from [16], which was shown to outperform original ESM2 in antibody specificity classification tasks. Both model types were trained and tested on identical datasets per fold. Statistical significance of AUROC improvement post-fine-tuning was assessed using a paired t-test (ns: **p** > 0.05, *: **p** ≤ 0.05, **: **p** ≤ 0.01).

As a performance baseline for specificity prediction, we evaluated the nested cross-validation performance of SVM model on the embeddings from the four pre-trained antibody language models as well as the original ESM2 protein language model for SARS-CoV-2 spike protein (Fig 2A and S2 Table) and influenza hemagglutinin (Fig 2C and S3 Table) specificity prediction. We used different sequence inputs to generate the embeddings, including paired full length (FULL HL), full-length heavy chain (FULL H), paired CDR3 (CDR3 HL), and CDR3 heavy chain (CDR3 H). Consistent with previously reporting [31], the performance improves as we include longer sequences of the receptors for each antibody language model (FULL HL> FULL H> CDR3 HL> CDR3 H). For the full-length paired sequence input, ft-ESM2 performs the best across the language models for most of the evaluation metrics with an average CV test AUROC of 0.88 for S protein and 0.86 for HA.

We fine-tuned the four antibody language models by training the last three layers of the pre-trained model along with sequence classification head to predict the specificity of SARS-CoV-2 spike protein (Fig 2B and S4 Tab**le**) and influenza hemagglutinin (Fig 2D and S5 Tab**le**) using the full-length paired BCR sequences and evaluated the performance of fine-tuning on the test set by the same data split using the four-fold cross validation procedure as the pre-trained embedding procedure. For both antigens, we noticed an increase in the AUROC for all CV folds for fine-tuned classifiers compared with pre-trained embedding classifiers. We performed paired Wilcoxon-rank sum tests to examine whether the increases are significant and found that the increases are significant for all models except the ft-ESM2 after fine-tuning for S protein classification.

### Fine-tuning increases model attention at the CDR regions

**Fig 3:**
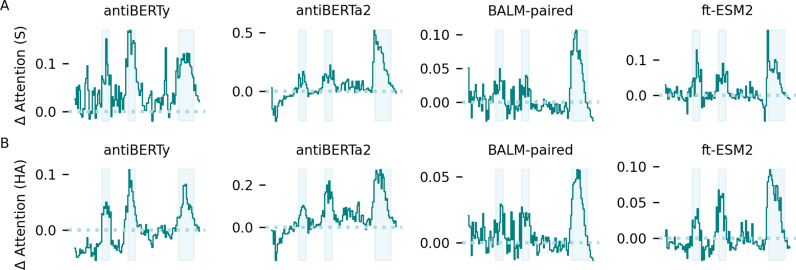
Change in average intra-heavy chain attention after fine-tuning. Attention activations were extracted from pre-trained and fine-tuned language models for 50 randomly selected antibodies specific for SARS-CoV-2 spike protein **(A)** and influenza HA **(B)**, respectively. The intra-heavy chain attention activations were averaged across heads and layers for each position on the heavy chain. Differences in average attention activations before and after fine-tuning for the last three layers were computed. The x-axis represents the position along the heavy chain. The solid line indicates the mean change in average attention activations across the 50 antibodies. The gray background indicates the regions spanned by CDR of the antibodies. The dotted line represents the mean of the difference in attention activations.

Previous studies [10,16,33] have shown that protein language models trained on antibody sequences with the masked language model objective have increased self-attention activations on the locations of long-range structural contacts or functionally important regions for binding. To evaluate the effect of supervised specificity fine-tuning on the antibody language model self-attention activations [17], we randomly selected fifty antibodies specific for SARS-CoV-2 S protein and influenza HA from the training dataset, and computed the average intra-chain attention along the antibody heavy and light sequences across the last three layers of the four antibody language models before and after fine-tuning. We took the differences of the average intra-chain attention between the fine-tuned and pre-trained model and found an increase in average attention activations across all four models after fine-tuning in positions corresponding to the CDR regions, especially the CDR3 regions (**Figs 3**, S5 and S6). The attention patterns are still more consistent for the same models across different fine-tuning tasks than between different models fine-tuned on the same antigen, which suggests that general sequence features learned during pre-training are retained even as models adapt to task-specific fine-tuning.

### Fine-tuned specificity classifiers capture changes in repertoire following vaccination

To further evaluate if the fine-tuned language model classifiers capture specificity information, we applied the classifiers to two single-cell BCR repertoire datasets measuring immune response to SARS-CoV-2 vaccination and influenza vaccination [30,32].

**Fig 4:**
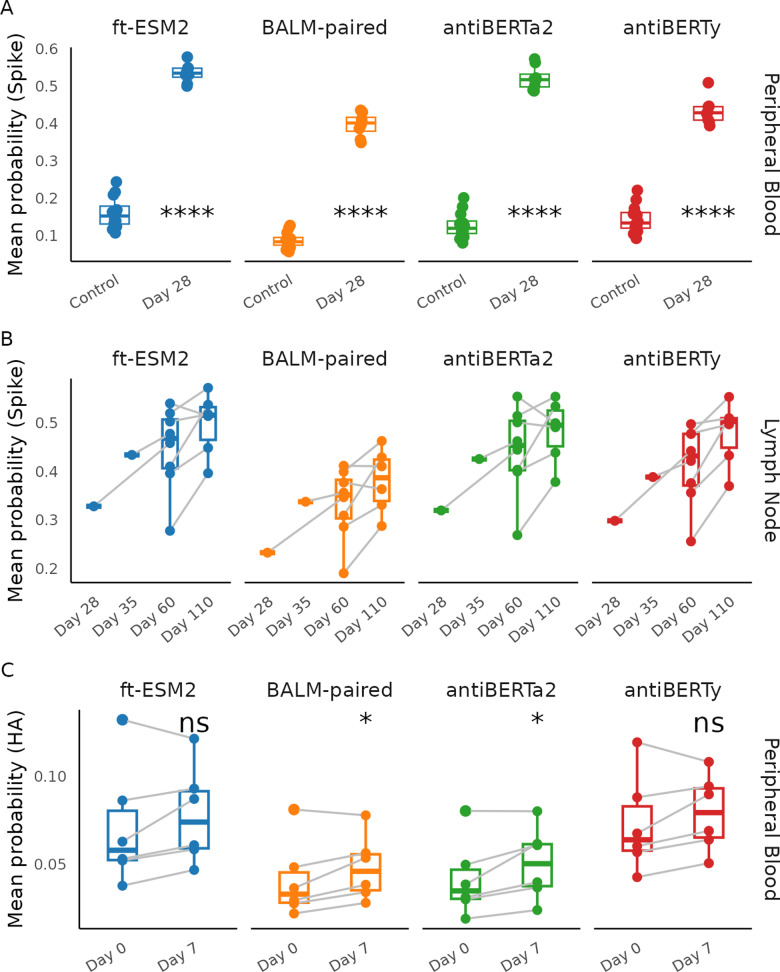
Application of the fine-tuned language model-based classifiers to vaccine response repertoire datasets. (A) Mean predicted probability of SARS-CoV-2 S protein binders by fine-tuned language model spike protein classifier of the receptors from peripheral blood samples 28 days after SARS-CoV-2 vaccination (Day 28), compared with the pre-pandemic repertoire datasets (Control). Samples from the same donor were connected by lines. Paired Wilcoxon rank sum test was used to obtain the significance level of the increase in mean predicted probability at day 7 (ns: **p** > 0.05, *: **p** <= 0.05, **: **p** <= 0.01, ***: **p** <= 1e-3, ****: **p** <= 1e-4). (B) Mean predicted probability of S protein binders applied to lymph node repertoires 28, 35, 60, 110 days after SARS-CoV-2 vaccination. (C) Mean predicted probability of influenza HA binders by language model fine-tuned on HA classification task applied to peripheral blood repertoires before and seven days after influenza vaccination.

In the SARS-CoV-2 mRNA vaccination dataset, eight donors had samples taken from two different tissues at various time points after SARS-CoV-2 vaccination: peripheral blood plasmablasts taken one week after the second immunization (Day 28) and axillary lymph node samples taken one to fifteen weeks after vaccination (Day 28, Day 35, Day 60, Day 110). We first applied the fine-tuned language model S protein classifiers on individual sequences of the SARS-CoV-2 vaccination dataset and a control peripheral blood dataset, which were taken pre-pandemic and assumed to have low level of S protein-specific sequences, to see if the spike protein classifiers can capture the immune response. We excluded any sequences within the same clone of the sequences in our training dataset to prevent data leakage and only kept one sequence from each clone to weight each clone equally. The SARS-CoV-2 vaccination repertoires are similar in distribution of gene usage, CDR3 length and somatic hypermutation frequency with the control samples (S2 F**ig**). We then averaged the predicted class probability from the spike protein classifiers. We tested the difference in the mean predicted probability of binding to spike protein between the peripheral blood plasmablast data and the control datasets using a Wilcoxon rank-sum test and found a significantly higher mean predicted probability of binding to S protein for the samples after SARS-CoV-2 vaccination repertoires (Fig 4A), which matches with the plasmablast response after vaccination. Similarly, we applied the spike protein classifiers to the lymph node repertoires after SARS-CoV-2 vaccination and computed the mean predicted probability. We found a persistent level of the mean predicted probability across the timepoint, which is also consistent with the robust and persistent germinal center response observed after two doses of the SARS-CoV-2 vaccination (Fig 4B).

We used the same criterion to process the influenza vaccination repertoire datasets, which consisted of six influenza vaccine-responsive donors with peripheral blood samples taken at pre-vaccination (Day 0) and seven days post-vaccination (Day 7) timepoints for paired BCR heavy and light chain sequencing. We similarly applied the fine-tuned language model classifiers to individual sequences within the repertoires to compute predicted probability of individual sequences of binding to HA and average the predicted probability for each sample (Fig 4C). We performed Wilcoxon-rank sum tests between the two timepoints, and found an increase in average predicted class probability at Day 7 for all four fine-tuned models, with antiBERTa2 and BALM-pair significantly increased by paired Wilcoxon-rank sum tests, which is consistent with the vaccine response observations based on the HAI titer [30].

## Discussion

In this study, we investigated the efficacy of supervised fine-tuning on pre-trained antibody language models to improve specificity prediction to two key antigens, the SARS-CoV-2 spike protein and influenza hemagglutinin. We established a performance baseline using nested cross-validation of SVM models on pre-trained language model embeddings, showing improved performance with full-length input receptor sequences as opposed to just CDR regions. We compared the performance of fine-tuned models to supervised classifiers trained on embeddings from the same pre-trained language models and found that fine-tuning the language models led to enhanced specificity prediction. Previous studies suggest that fine-tuning can yield improvements with as few as 1,000 high quality examples [34]. In our case, we observed performance gains in our HA fine-tuning task using a relatively small dataset of 5,000 sequences. Parameter-efficient fine-tuning methods like LoRA [35] could further reduce data requirements by adapting fewer parameters, making fine-tuning more accessible with limited resources. Additionally, we observed increased attention at the CDR regions after fine-tuning, indicative of the models capturing relevant features for antigen specificity. Furthermore, we applied the fine-tuned classifiers to longitudinal paired BCR repertoire data related to influenza and SARS-CoV-2 vaccination, showing their ability to capture changes in repertoire following vaccination, as evidenced by shifts in predicted binding probabilities.

While our study provides valuable insights into the effectiveness of fine-tuning pre-trained antibody language models for antigen specificity prediction, several limitations should be acknowledged. Firstly, the performance of the fine-tuned models may be influenced by the size and composition of the training datasets as well as the inclusion of non-ground-truth negative class sequences, which could affect the generalizability of our findings to different datasets on spike protein and influenza HA specificity tasks [36,37]. Additionally, fine-tuning on unrelated protein sequence types could serve as a control experiment to examine which aspects of task-specific fine-tuning are causally linked with the observed prediction accuracy gains. Secondly, our evaluation focused primarily on two specific antigens, the SARS-CoV-2 spike protein and influenza hemagglutinin. Further study is needed to examine broader applicability of our conclusions to other antigens or biological contexts. Furthermore, since our classifier was trained without variant or epitope annotations, it might not capture features related to variant-specific binding. Future work incorporating strain-level metadata or epitope-binding annotations could help distinguish between conserved and variant-specific binding patterns by leveraging approaches such as multi-task learning, where the model simultaneously predicts antigen specificity and epitope-level binding features, enabling better generalization across diverse antigen variants. Additionally, the interpretation of attention activations changes after fine-tuning may be constrained by the complexity of attention mechanisms in language models since this extracted pattern may not have a straightforward mapping with the interactions between amino acid residues. Future studies on ground truth data are needed to further examine the utility of attention patterns for interpretability. More generalizable methods, including linguistics-inspired experimentation and grammatical inference, has been suggested as potential approaches to extracting sequence-function rules that the model has learned [38].

In summary, our study demonstrates the efficacy of fine-tuning pre-trained antibody language models to enhance specificity prediction. We established performance baselines and observed improved prediction accuracy with fine-tuned models, particularly in capturing changes in repertoire following vaccination. The findings give insights for further studies to advancing our understanding of antigen specificity prediction applications using antibody language models.

## Materials and methods

### Models

We downloaded the following four pre-trained antibody language models, with the size parameters listed in Table 1. Details in model specifications are available in S1 Text and S1 Tab**le**.

**antiBERTy** [9]: Pre-trained using the BERT architecture on 588 million unpaired antibody heavy and light chain sequences from multiple species using a masked language modeling (MLM) objective.

**antiBERTa2** [11]: Based on the RoFormer architecture, pre-trained with 1.54 billion unpaired and 2.9 million paired human antibody sequences with MLM objective.

**BALM-paired** [16]: Developed using a RoBERTa-large architecture trained on 1.34 million paired antibody sequences with MLM objective.

**ft-ESM2** [16]: Based on 650-million parameter ESM2 (Evolutionary Scale Modeling) model [19], fine-tuned with 1.34 million paired antibody sequences with MLM objective.

We have also included one-hot encoding and 3-mer frequency transformed by TF-IDF, to serve as a baseline performance comparison for the pre-trained model embeddings.

### Data sources

We collected antibody sequences with specificity labels to influenza HA protein and SARS-CoV-2 Spike protein from public sources, as listed in Table 2.

*Influenza HA-specific sequences* We extracted the paired-chain antibody sequences with influenza HA proteins binding/non-binding labels from public datasets [32,26,29], which consisted of 3,221 sequences binding to various HA proteins as well as 706 were non-binding. To balance the labels, we sampled additional vaccine non-responsive sequences from six pre-vaccination repertoires in [30] as additional negative controls. The distribution in V, J gene usage and CDR3 length is similar between the negative controls and the positive sequences (S1 F**ig**). In total, 6,424 receptors were available, with 3,221 binding (50.1%).

*SARS-CoV2 spike protein-specific sequences* We used the antibody sequences dataset we previously curated with binding labels to SARS-CoV-2 [31].

*Repertoire data* We collected additional single-cell paired-chain repertoire data from [30], which had peripheral blood samples collected before and seven days after influenza vaccination, as well as [32], which had both peripheral blood and lymph node samples taken from 28, 35, 60 and 110 days after SARS-CoV-2 vaccination.

### Receptor specificity prediction using pre-trained language model embedding

To establish a baseline performance for the four language models in predicting specificity to the SARS-CoV-2 spike protein and influenza hemagglutinin proteins, we trained supervised models using the pre-trained model embedding as input. The process involved concatenating each pair of BCR heavy and light chain sequences, separated by two [CLS] tokens, and feeding them into each pre-trained antibody language model to obtain the output from the last hidden layer. Then, utilizing this embedding as input, we trained separate support vector machine (SVM) classifiers to predict the binary binding status for each antigen from each pre-trained model embedding.

Specifically, we employed sklearn SVM with an RBF kernel and implemented nested cross-validation to split the data into training, validation, and test sets, ensuring non-overlapping donors and preserving class percentage with sklearn.model_selection.StratifiedGroupKFold. Three inner loops and four outer loops were utilized for hyperparameter search on the validation set and to compute test set performance, respectively. During hyperparameter search, we conducted a grid search over the regularization parameter C of SVM, ranging from 0.01 to 100, and selected the optimum value based on the validation set AUROC score.

Evaluation of the test set performance included metrics such as AUROC, weighted-average F1 score, precision, recall, average precision score, balanced accuracy, and Matthews correlation coefficient. Finally, we chose the regularization parameters that yielded the best validation AUROC across nested CV outer folds and trained the final classifier using all available binding data.

### Supervised Fine-tuning of language models for receptor specificity prediction

We fine-tuned the last three layers of each of the four language models to predict the binary binding or non-binding status to either the SARS-CoV-2 spike protein or influenza hemagglutinin proteins. We assessed the performance of this fine-tuning by using the same cross-validation train-test data split employed in the embedding SVM approach for direct comparison. For each training dataset, we separated out a validation set (33%) to determine the optimum epoch. To fine-tune each language model, we instantiated a sequence classification model using transformers.AutoModelForSequenceClassification) and initialized it with the pre-trained weights for each model in Table 1. We trained each classification model with a learning rate of 1e-5, a batch size of 64 for 30 epochs, and selected models from epochs with the best validation AUROC to evaluate the test set performance by AUROC, weighted-average F1 score, precision, recall, average precision score, balanced accuracy, and Matthews correlation coefficient. We determined the epoch that yielded the best average validation AUROC across outer folds and trained the final classifier using all available binding data. All models were fine-tuned on a single NVIDIA RTX A5000 GPU.

### Applying sequence specificity classifiers to repertoires

To determine whether the classifier effectively identifies BCR specificity, we applied the classifiers to paired-chain BCR repertoire data from vaccinations against SARS-CoV-2 and influenza. In processing these datasets, we used the immcantation Change-O pipeline [39] to cluster BCR sequences into clonal groups. To prevent data leakage, we excluded sequences from the repertoires that belonged to the same clone as those used in training the specificity classifiers. Additionally, to minimize the confounding effects of clonal expansion, we retained only one sequence from each clone. For each sequence, we calculated the predicted class probability of binding to a given antigen and then computed the average of these predicted probabilities for each repertoire.

## Supporting Information

S1 Text Pre-trained language model specifications. (DOCX)

S1 Table Training dataset of the pre-trained language models. (XLSX)

S2 Table Prediction performance of pre-trained antibody language model embeddings and control models for SARS-CoV2 S protein specificity prediction task. Supervised SVM models were trained on language model embeddings of antibody sequences to predict the binding status of the antibody to Influenza HA. Nested cross-validation was used to evaluate the prediction performance and the median Area under Receiver Operating Characteristics (AUROC), average weighted F1 score (F1), Precision, Recall, Average Precision Score (AP), Balanced Accuracy Score, Matthew’s correlation coefficient (MCC) across the outer loops were shown in the table.
(XLSX)

S3 Table Prediction performance of pre-trained antibody language model embeddings and baseline models for influenza HA specificity prediction task. Supervised SVM models were trained on language model embeddings of antibody sequences to predict the binding status of the antibody to Influenza HA. Nested cross-validation was used to evaluate the prediction performance and the median Area under Receiver Operating Characteristics (AUC), average weighted F1 score (F1), Precision, Recall, Average Precision Score (AP), Balanced Accuracy Score, Matthew’s correlation coefficient (MCC) across the outer loops were shown in the table.
(XLSX)

S4 Table Performance of language models on SARS-CoV2 S protein specificity prediction task after fine-tuning. (XLSX)

S5 Table Performance of language models on influenza HA specificity prediction task after fine-tuning. (XLSX)

S1 Fig Distribution of biological properties between the sequences with HA binding labels and sampled non-binding control sequences. (A) Distribution of gene usage of sequences with HA binding labels (Labelled) and control sequences sampled from the vaccine non-responsive cells repertoires (Control). (B) Distribution of CDR3 length and somatic hypermutation frequency between labelled and control sequences. Abbreviations: Jfam: J gene family, Vfam: V gene family, mu: somatic hypermutation frequency, H: heavy chain, L: light chain.
(TIF)

S2 Fig Distribution of biological properties between the sequences from the SARS-CoV-2 vaccination repertoire data (Kim 2022) and control samples (Wang M. 2023). **(A)** Distribution of gene usage of sequences between the two datasets. **(B)** Distribution of CDR3 length and somatic hypermutation frequency between the two datasets. Abbreviations: Jfam: J gene family, Vfam: V gene family, mu: somatic hypermutation frequency, H: heavy chain, L: light chain.
(TIF)

S3 Fig Validation loss for fine-tuning antibody language models for SARS-CoV-2 spike protein binding prediction. Four antibody language models were fine-tuned for 30 epochs for SARS-CoV-2 spike protein binding predictions. Each horizontal panel shows the validation loss for one of the folds in a 4-fold cross validation procedure. The red dot represents the final model selected with the best validation AUROC and used to evaluate the performance on the test dataset.
(TIF)

S4 Fig Validation loss for fine-tuning antibody language models for influenza hemagglutinin binding prediction. Four antibody language models were fine-tuned for 30 epochs for influenza hemagglutinin binding predictions. Each horizontal panel shows the validation loss for one of the folds in a 4-fold cross validation procedure. The red dot represents the final model selected with the best validation AUROC and used to evaluate the performance on the test dataset.
(TIF)

S5 Fig Change in intra-light chain attention after specificity fine-tuning for (A) SARS-CoV2 spike protein and (B) influenza HA. (TIF)

S6 Fig Intra-heavy chain attention before and after fine-tuning across embeddings and antigens. Comparison of model attention before (pre-trained, coral) and after (fine-tuned, teal) fine-tuning across four different embeddings for (A) SARS-CoV2 spike protein and (B) influenza HA.
(TIF)
