# Supplementary material for: Supervised fine-tuning of pre-trained antibody language models improves antigen specificity prediction
Source: PLoS Comput Biol. 2025 Mar 31;21(3):e1012153. doi: 10.1371/journal.pcbi.1012153 (PMC12013870; doi:10.1371/journal.pcbi.1012153)
Supplement: S1 Text — (DOCX) [file pcbi.1012153.s001.docx]

**S1 Text. Pre-trained language model specifications**

A summary of the model architecture, training procedure, as well as the training data is shown in detail below:

**AntiBERTy [1]**: This model uses the BERT transformer encoder architecture, which is known to capture long-range dependencies in sequences [2], and is trained on 588 million non-redundant antibody sequences derived from the Observed Antibody Space database [3], which includes heavy and light chains from six species. The model comprises 8 layers with 8 attention heads per layer, a hidden dimension of 512, and a feedforward dimension of 2048, resulting in approximately 26 million trainable parameters. Training was performed over 8 epochs using 558 million sequences from multiple species (human, mouse, rat, camel, rabbit, rhesus) for training and 1 million for evaluation, parallelized across four NVIDIA A100 GPUs over 10 days. AntiBERTy has been shown to effectively encode the sequence-structure-function relationships in antibodies [4] making it a suitable candidate for specificity prediction.

**AntiBERTa2 [5]**: Based on the RoFormer architecture, a variant of the RoBERTa model, this model incorporates rotational position encodings, which improve the model's ability to learn sequence patterns [3]. AntiBERTa2 was pre-trained on a combination of 1.54 billion unpaired and 2.9 million paired human antibody sequences using a masked language modeling (MLM) objective. The model has 12 layers, each with 12 attention heads, a hidden dimension of 768, and a feed-forward dimension of 3072, totaling 86 million parameters. The training involved 225,000 steps over three epochs, using a batch size of 96 across eight NVIDIA V100 GPUs, with a peak learning rate of 1e-4. AntiBERTa2's extensive pre-training on a large and diverse dataset enhances its generalization capabilities, making it well-suited for fine-tuning tasks such as antigen specificity prediction.

**BALM-paired [6]**: This model uses a RoBERTa-large architecture, trained on 1.34 million paired antibody sequences from healthy adult human donors. BALM-paired consists of 24 layers with 16 attention heads per layer, a hidden size of 1,024, and an intermediate size of 4,096. The input sequences, concatenated heavy and light chains separated by a special token, were padded to a maximum length of 512. The model was trained for 500,000 steps (approximately 100 epochs) with a peak learning rate of 4e-4, utilizing eight NVIDIA A100 GPUs over 5 days. BALM-paired's training on paired sequences allows it to capture the interactions between heavy and light chains, which are essential for predicting antibody-antigen binding.

**ft-ESM2 [6]**: This model fine-tunes the 650-million parameter ESM-2 model, based on the RoBERTa architecture, with 33 layers and 20 attention heads per layer. It was fine-tuned on 1.34 million paired antibody sequences, with inputs consisting of concatenated heavy and light chain sequences padded to a maximum length of 320. The fine-tuning process lasted for 150,000 steps using a peak learning rate of 4e-4, performed on eight NVIDIA A100 GPUs over 7 days. The ESM-2 model has demonstrated high accuracy in protein sequence modeling tasks [7]. Fine-tuning this model on antibody sequences aims to leverage its pre-trained knowledge while adapting it to the specific task of antigen specificity prediction.

**References**

1. Ruffolo JA, Gray JJ, Sulam J. Deciphering antibody affinity maturation with language models and weakly supervised learning. arXiv. 2021; 2112.07782. doi:10.48550/arXiv.2112.07782

2. Devlin J, Chang M-W, Lee K, Toutanova K. BERT: Pre-training of Deep Bidirectional Transformers for Language Understanding. arXiv. 2018; 1810.04805. doi:10.48550/arXiv.1810.04805

3. Olsen TH, Boyles F, Deane CM. Observed Antibody Space: A diverse database of cleaned, annotated, and translated unpaired and paired antibody sequences. Protein Science. 2022;31: 141–146. doi:10.1002/pro.4205

4. Ruffolo JA, Chu L-S, Mahajan SP, Gray JJ. Fast, accurate antibody structure prediction from deep learning on massive set of natural antibodies. Nat Commun. 2023;14: 2389. doi:10.1038/s41467-023-38063-x

5. Barton J, Galson JD, Leem J. Enhancing Antibody Language Models with Structural Information. arXiv. 2023; 2023–12. doi:10.1101/2023.12.12.569610

6. Burbach SM, Briney B. Improving antibody language models with native pairing. arXiv. 2023; 2308.14300. doi:10.48550/arXiv.2308.14300

7. Lin Z, Akin H, Rao R, Hie B, Zhu Z, Lu W, et al. Evolutionary-scale prediction of atomic-level protein structure with a language model. Science. 2023;379: 1123–1130. doi:10.1126/science.ade2574
