# Supplementary figures and images for: Supervised fine-tuning of pre-trained antibody language models improves antigen specificity prediction
Source: PLoS Comput Biol. 2025 Mar 31;21(3):e1012153. doi: 10.1371/journal.pcbi.1012153 (PMC12013870; doi:10.1371/journal.pcbi.1012153)

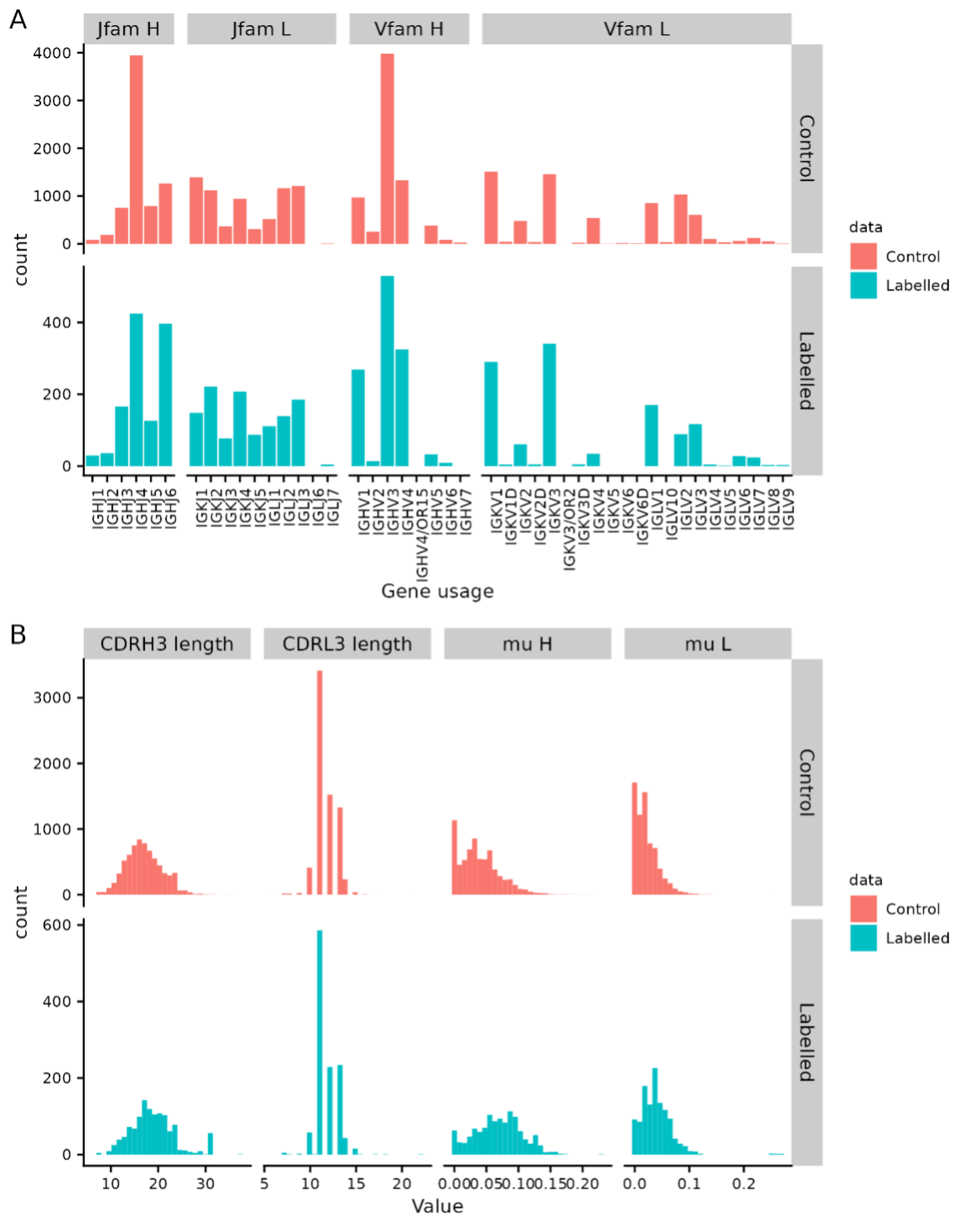

Supplement: S1 Fig — (A) Distribution of gene usage of sequences with HA binding labels (Labelled) and control sequences sampled from the vaccine non-responsive cells repertoires (Control). (B) Distribution of CDR3 length and somatic hypermutation frequency between labelled and control sequences. Abbreviations: Jfam: J gene family, Vfam: V gene family, mu: somatic hypermutation frequency, H: heavy chain, L: light chain. (TIF) [file pcbi.1012153.s007.tif]

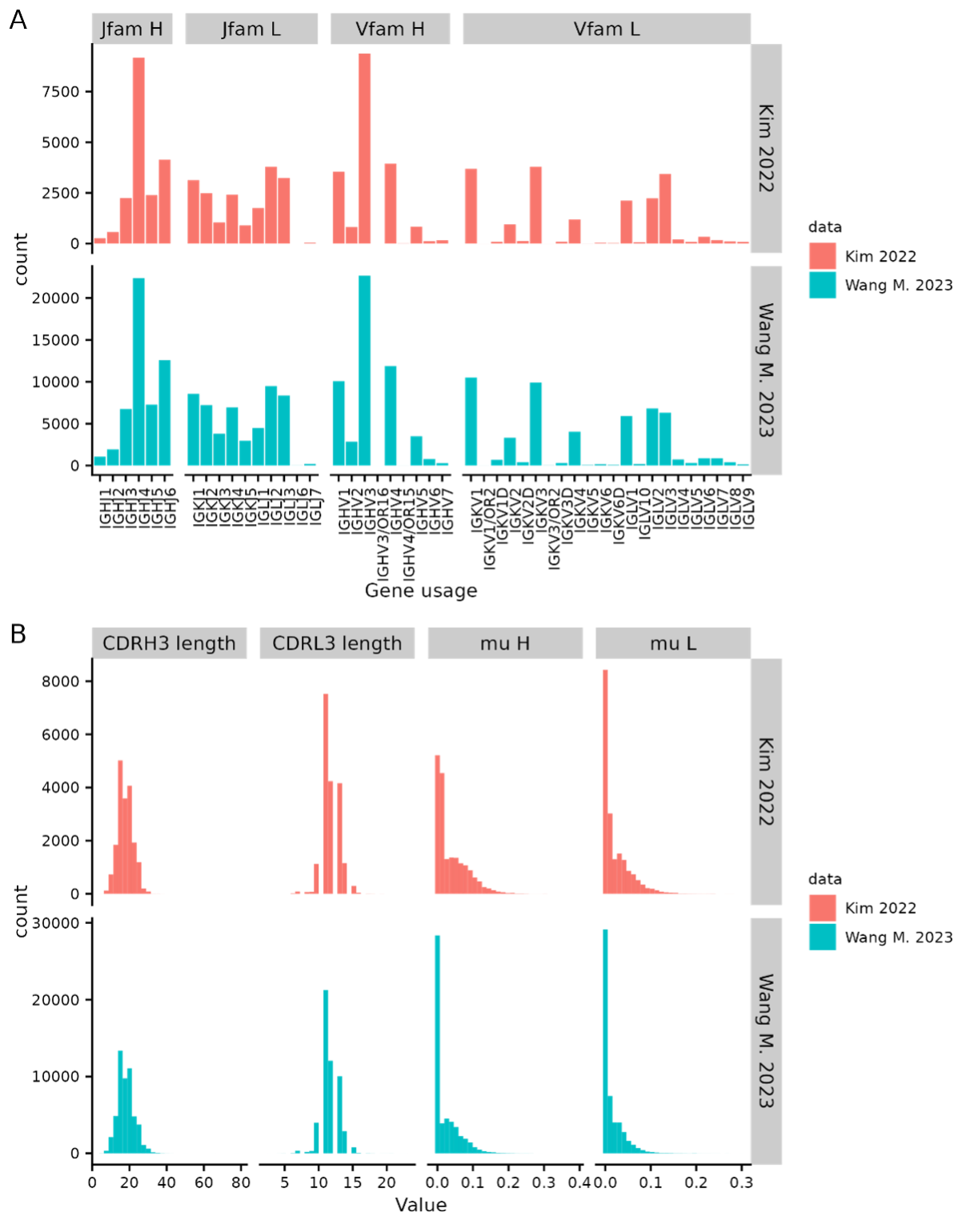

Supplement: S2 Fig — (A) Distribution of gene usage of sequences between the two datasets. (B) Distribution of CDR3 length and somatic hypermutation frequency between the two datasets. Abbreviations: Jfam: J gene family, Vfam: V gene family, mu: somatic hypermutation frequency, H: heavy chain, L: light chain. (TIF) [file pcbi.1012153.s008.tif]

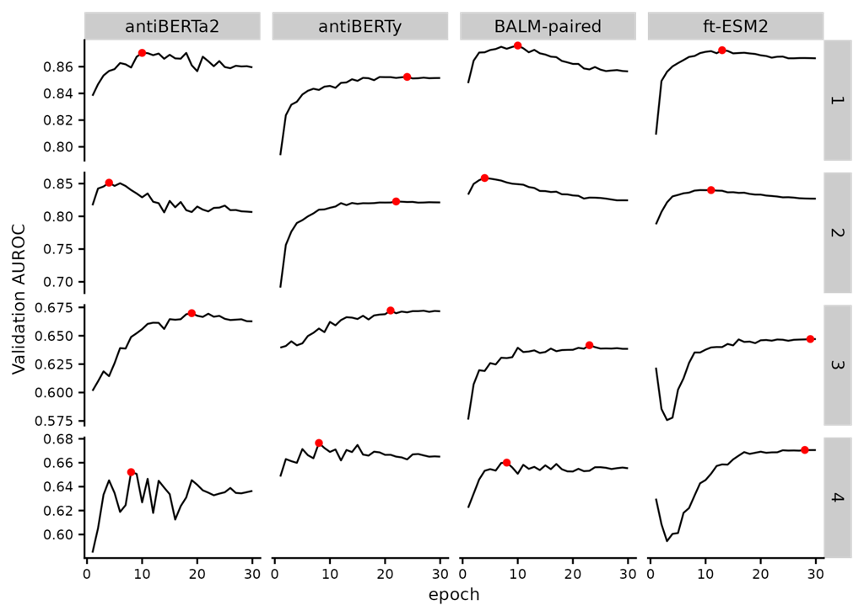

Supplement: S3 Fig — Four antibody language models were fine-tuned for 30 epochs for SARS-CoV-2 spike protein binding predictions. Each horizontal panel shows the validation loss for one of the folds in a 4-fold cross validation procedure. The red dot represents the final model selected with the best validation AUROC and used to evaluate the performance on the test dataset. (TIF) [file pcbi.1012153.s009.tif]

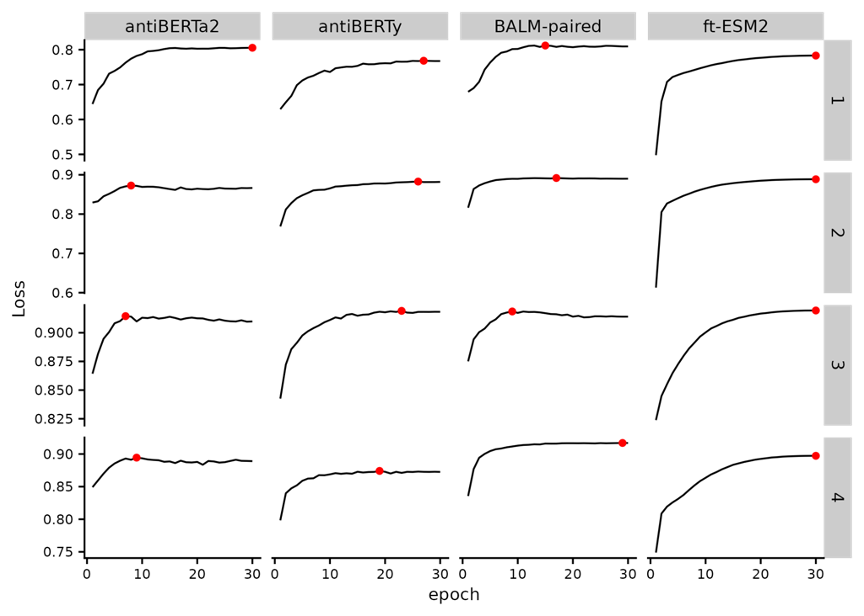

Supplement: S4 Fig — Four antibody language models were fine-tuned for 30 epochs for influenza hemagglutinin binding predictions. Each horizontal panel shows the validation loss for one of the folds in a 4-fold cross validation procedure. The red dot represents the final model selected with the best validation AUROC and used to evaluate the performance on the test dataset. (TIF) [file pcbi.1012153.s010.tif]

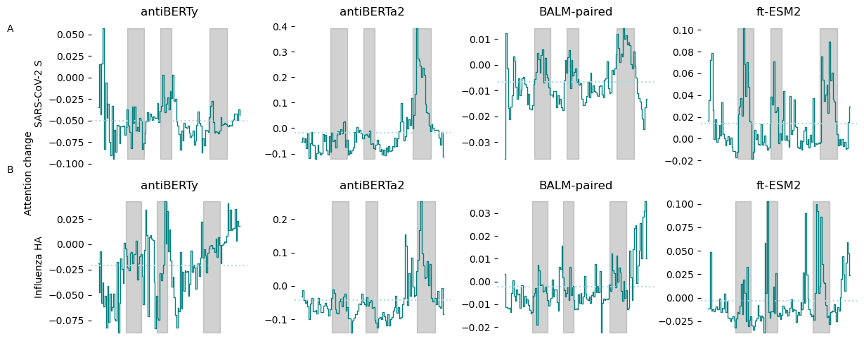

Supplement: S5 Fig — (TIF) [file pcbi.1012153.s011.tif]

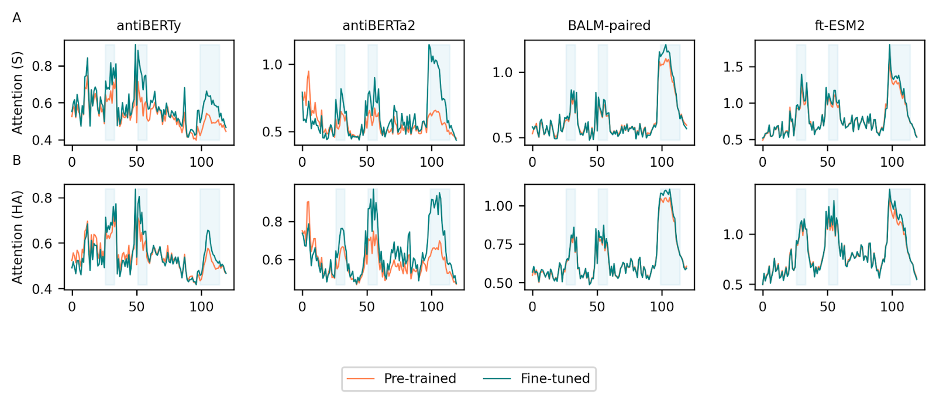

Supplement: S6 Fig — Comparison of model attention before (pre-trained, coral) and after (fine-tuned, teal) fine-tuning across four different embeddings for (A) SARS-CoV2 spike protein and (B) influenza HA. (TIF) [file pcbi.1012153.s012.tif]
